# Multitarget docking and molecular enumeration reveal *DdpMPyPEPhU* as a potent modulator of cell cycle, glucocorticoid, and estrogen signalling in breast cancer

**DOI:** 10.1371/journal.pone.0344028

**Published:** 2026-03-23

**Authors:** Shaban Ahmad, Sahar Qazi, Nagmi Bano, Rahis Uddin, Pramod Kumar Gautam, Khalid Raza

**Affiliations:** 1 Department of Computer Science, Jamia Millia Islamia, New Delhi, India; 2 Department of Chemistry, Jamia Millia Islamia, New Delhi, India; 3 Department of Biochemistry, All India Institute of Medical Sciences, New Delhi, India; Atma Ram Sanatan Dharma College University of Delhi, INDIA

## Abstract

Breast cancer is one of the most prevalent cancers worldwide, ranked as the second most diagnosed cancer and the fourth leading cause of cancer-related deaths. Despite the availability of FDA-approved therapies, limitations such as drug resistance and off-target effects highlight the need for novel, multitargeted therapeutic agents. In this study, we aimed to identify and design an in-silico promising multitarget drug for breast cancer by simultaneously targeting three critical proteins: Glucocorticoid Receptor, Estrogen Receptor-alpha (ER-alpha), and Cyclin-Dependent Kinase 2 (CDK2). FDA-approved drugs corresponding to these targets were initially subjected to multitarget molecular docking to evaluate their binding affinities. Based on this screening, the 15 highest-ranking ligands were selected and underwent molecular enumeration, resulting in the generation of 14,750 novel derivative compounds. The re-docking identified 1-((R)-2,3-dihydroxypropyl) −3-(3-((R)-1–5-methyl-1H-pyrrolo [2,3-b]pyridin-3-yl)ethyl)phenyl) urea (DdpMPyPEPhU) (Patent No. 202024101028.0) as a promising multitarget candidate. The compound exhibited enhanced binding pocket engagement through numerous stabilising interactions, including hydrogen bonds, π-π stacking, and π-cation interactions, with high docking scores (–14.869 to –4.57 kcal/mol) and favourable Molecular Mechanics Generalised Born Surface Area (MM-GBSA) energies (–72.32 to –11.97 kcal/mol). Comparative docking and pharmacokinetic analyses with standard drugs Lapatinib and Tamoxifen indicated better drug-like properties and pharmacokinetic advantages for DdpMPyPEPhU. Additional validation using Density Functional Theory (DFT) optimisation, 5 ns WaterMap analysis, and 250 ns molecular dynamics simulations under neutralised conditions confirmed structural stability and strong intermolecular interactions, supported by binding free energy calculations. Overall, our computational findings suggest that DdpMPyPEPhU is a promising therapeutic candidate for breast cancer, providing a rational basis for further experimental evaluation.

## 1. Introduction

Cancer is the uncontrolled and unchecked growth of abnormal cells in the body, which develop like other cells. However, they continue to grow and can form a mass, eventually becoming tumours [1]. Cancer arises from the transformation of normal cells into tumour cells in various stages that progress from pre-cancerous to malignant [2]. Since cells are present in every part of our body, cancer can also grow in all parts of our body. Also, according to the World Health Organisation (WHO), in 2020, cancer caused more than 10 million deaths [3,4]. Therefore, identifying the histological type of cancer and the respective protein biomarkers is crucial for substantial therapy. Due to the considerable strangeness in the molecular-biological distinction of cancer histological types, it is impossible to ascertain without knowing the nature and origin of malignant cells, which display specific protein biomarkers in the bloodstream [5,6]. The disease can begin anywhere in the human body, consisting of trillions of cells. Human cells continuously develop and replicate to form new cells as the body needs. Benign tumours do not spread extensively, and if cells are removed, tumours usually do not recur, although malignant cancers sometimes do [7]. Benign tumours can now and again be enormous, however. Some can cause genuine symptoms or be dangerous, such as benign tumours in the brain [8]. Cancer is the leading cause of death globally, and the most common death-causing cancer in 2020 was the breast, lung, colon and rectum, prostate, skin, and stomach [4]. Indeed, even with excess deaths and research, there is no cure for cancer or even a medicine that is supposed to be effective and accurate [9,10]. The drawbacks of these methods include the toxicity and side effects of utilising traditional chemicals to treat cancer [11]. Finding novel, effective medications to prevent and treat this disease with few side effects is necessary, given the failure of existing chemotherapeutic treatments, and plants can be a crucial source of these promising compounds. Plants, made of natural ingredients, have effectively treated many ailments, including cancer. The plant kingdom is the source of approximately 60% of anticancer medications and represents an entirely natural system for these medications [12]. Despite extensive research into cancer and its molecular pathways, target identification, and therapy development, cancer remains the primary cause of mortality in economically developed and developing nations [13]. Cyclooxygenase-2 (COX-2) is linked to carcinogenesis, specifically angiogenesis and breast cancer, and predicts tumour progression. The physiological mechanism of COX-2 states that two isoenzymes, such as COX-1 and COX-2, produce prostaglandins from arachidonic acid [14]. Although many research groups and institutions are working towards curing cancer, there is no cure because of resistance and multiple factors involved in cancer development, and the multitargeted drug designing approach comes to mind [15,16].

Breast cancer remains one of the leading causes of cancer-related mortality among women worldwide, largely due to therapeutic resistance and molecular heterogeneity across subtypes [1,2]. A major limitation of current therapies is their reliance on single-target mechanisms, which often fail to suppress compensatory signaling pathways that sustain tumour survival and progression. Consequently, multitarget drug discovery has emerged as a powerful strategy to overcome resistance and improve long-term therapeutic efficacy [17]. Breast cancer is a hormone-dependent malignancy in which estrogen receptor-α (ER-α) plays a central role in regulating transcriptional programs that drive proliferation, survival, and endocrine therapy resistance. ER-α [18] activation stimulates oncogenic signaling pathways, including PI3K/AKT/mTOR and MAPK/ERK, while crosstalk with growth factor receptors such as HER2 further enhances tumour aggressiveness. In parallel, dysregulation of cell-cycle control through Cyclin-Dependent Kinase 2 (CDK2) [19] promotes uncontrolled DNA replication and tumour growth. CDK2 activity frequently cooperates with ER-α signaling, linking hormone-driven transcription with cell-cycle progression.

In addition to ER-α, CDK2, and the glucocorticoid receptor (GR) [20], has emerged as a critical modulator of breast cancer biology. GR signaling supports tumour cell survival in triple-negative and endocrine-resistant breast cancers by suppressing apoptosis and enhancing stress-adaptation pathways. Moreover, functional crosstalk between GR and ER-α reshapes hormone-regulated transcription, while GR–CDK2 interactions influence cell-cycle checkpoints. Together, ER-α, CDK2, and GR form an interconnected regulatory network governing proliferation, survival, and therapeutic resistance in breast cancer. Therefore, simultaneous targeting of ER-α, CDK2, and GR represents a biologically and clinically justified multitarget strategy for disrupting key oncogenic pathways and overcoming resistance mechanisms.

Regulation of proliferation and cell cycle progression is mainly performed by cell cycle regulators such as Cyclin-dependent kinase 2 (CDK2), CDK4 and CDK6 [21]. When these proteins are not working properly, cells can grow uncontrollably, a common feature of cancer. Transcription factors like Estrogen Receptor-α (ERα) and p53 help to control which genes are switched on or off in cells. Changes in the activity of ERα and p53 can disrupt this process, leading to abnormal cell growth and cancer development [22,23,24]. In breast cancer, ERα plays a central role in hormone-driven signalling by binding estrogen and regulating downstream pathways such as PI3K/AKT/mTOR and MAPK/ERK, which promote cell survival, proliferation, and resistance to apoptosis [25,26]. Crosstalk between ERα and growth factor receptors like HER2 amplifies oncogenic signalling, driving aggressive tumour progression and endocrine resistance [24]. CDK2, in collaboration with cyclins E and A, regulates the G1/S transition and DNA replication. Hyperactivation of CDK2 accelerates cell cycle progression, often working in tandem with ERα signalling to enhance proliferation [27]. Additionally, CDK2 activity is modulated by upstream mitogenic signals through the Rb-E2F pathway, linking growth factor signalling to uncontrolled DNA synthesis in cancer cells [ 28,29,30]. In addition to ER-α and CDK2, the glucocorticoid receptor (GR) was selected as a third therapeutic target due to its emerging role in breast cancer progression, therapy resistance, and subtype-specific survival. GR signaling has been reported to promote survival pathways in triple-negative and endocrine-resistant breast cancers by suppressing apoptosis and enhancing stress-adaptation mechanisms. Moreover, GR–ER crosstalk influences hormone-driven transcriptional programs, while GR–CDK2 interactions modulate cell-cycle checkpoints. Therefore, simultaneous modulation of ER-α, CDK2, and GR provides a clinically relevant multitarget strategy for overcoming resistance and heterogeneity in breast cancer.

In this study, a comprehensive computational drug discovery pipeline was used to identify potential inhibitors of DdpMPyPEPhU against breast cancer receptors, Glucocorticoid Receptor, Estrogen Receptor-α, Cyclin-Dependent Kinase 2 (PDB IDs: 1NHZ, 3ERT, and 1DI8). Beginning with 316 FDA-approved drugs, we performed structure preparation using LigPrep and the OPLS-2004 force field, generating 897 stereoisomers. Multi-tiered molecular docking was conducted using high-throughput virtual screening (HTVS), standard precision (SP), and extra-precise (XP) protocols (100% coverage), followed by MM-GBSA (Molecular Mechanics/Generalised Born Surface Area) binding free energy calculations to validate binding affinities. Subsequently, automated reaction-based enumeration generated 14,750 compounds, which were prepared into 84,941 stereoisomers via LigPrep. Post-enumeration docking and rigorous filtering based on docking scores (*threshold: < −5.0*) [31] and MMGBSA results yielded 967 high-affinity compounds, from which 15 drugs were selected for advanced analysis. The top candidates underwent 250-nanosecond molecular dynamics (MD) simulations to assess binding stability, complemented by **Density Functional Theory (DFT)** calculations and Highest Occupied Molecular Orbital (HOMO) and Lowest Unoccupied Molecular Orbital (LUMO) analysis of DdpMPyPEPhU complexes to characterise energetically favourable binding configurations. This integrated computational workflow systematically screened, optimised, and validated potential therapeutic candidates against breast cancer targets. Furthermore, our novel compound *DdpMPyPEPhU* was compared to the existing FDA-approved control drugs to highlight its potential as a multitargeted, lead-like therapeutic drug against breast cancer.

## 2. Methods

**Fig 1:**
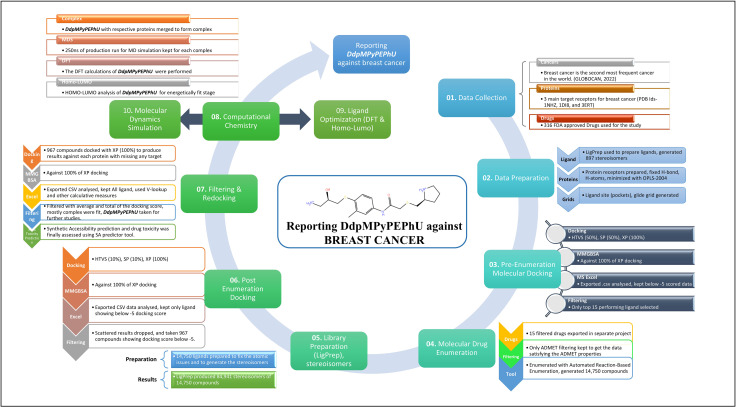
Comprehensive computational workflow for DdpMPyPEPhU drug discovery against breast cancer targets.

The methodology that leads us to identify the *DdpMPyPEPhU* as a potential candidate against breast cancer is complex and is displayed in Fig 1. We collected the 3D structures of crucial proteins and FDA-approved drugs against breast cancer to prepare them and docked them in the Schrödinger Maestro, followed by the molecular enumeration of most compounds to generate the novel candidate and recheck with re-docking and validation with other studies. Further, the detailed method is as follows-

### 2.1. Data collection and preparation

For collecting data for protein and ligand, we went through extensive literature mining to identify the crucial target protein data from the RCSB database (https://www.rcsb.org/) [32]. Further, the 316 drugs (ligand) data were identified from the FDA-approved (https://www.fda.gov/drugs/) list against these cancers and downloaded from the Drug Bank database (https://go.drugbank.com/) in the SDF, and a few of them were like vitamins and minerals provided to the patients as a supplement with the drug [33]. The complete data preparation is divided into two steps: one for ligand preparation and another for protein preparation. The detailed description is as follows-

#### 2.1.1. Ligand preparation.

The library’s three-dimensional (3D) coordinates were generated with the LigPrep tool in Schrödinger Maestro (https://schrodinger.com/) [34,35]. The OPLS4 force field was used while the ionisation state was kept at a pH of 7 (±2) [36]. The Epik with the metal-binding site, including the original state, was kept. Epik also approximates a penalty to quantify the likelihood of producing every stage resolution [37,38]. Further, the computational stereoisomers were kept for 32 per ligand while retaining the specified chirality, which resulted in 897 compounds from 316 drugs [34]. This setting was kept the same for each level of ligand preparation. A set of 316 FDA-approved drugs was retrieved from DrugBank based on three criteria: (i) approved clinical status, (ii) reported anticancer, endocrine-modulating, or kinase-inhibitory activity, and (iii) structural completeness for docking. No class-based filtering was applied to preserve chemical diversity for multitarget screening.

#### 2.1.2. Protein preparation.

The Protein Preparation Wizard in Schrödinger’s Maestro was used to prepare the proteins, a multilevel process [35,39,40]. The preprocessing includes the basic steps to download the PDB structure in the workspace, fill the gaps and missing chains, loops, or even amino acid levels using the Prime module, and generate the hetero state using Epik at pH 7 (±2) [37,38,41]. The review and modification steps are to review the proteins, delete the incomplete protein chain or the dimers, and rectify other problems with the protein to understand the bonding orders [42].

The rationale behind selecting the three receptors was mainly because these are renowned critical therapeutic targets in breast cancer, known for specific molecular mechanisms underlying tumour growth and progression. ER-α (PDB ID: 3ERT) is the most clinically significant target, expressed in approximately 70% of breast cancers, and serves as the primary target for endocrine therapy in hormone receptor-positive disease [27]. CDK2 (PDB ID: 1DI8) plays a crucial role in cell cycle regulation and is frequently dysregulated in breast cancer; its inhibition offers a therapeutic strategy for overcoming endocrine resistance and targeting highly proliferative tumours across multiple breast cancer subtypes. The glucocorticoid receptor (GR) (PDB ID: 1NHZ), while less conventional, has emerged as an important modulator of breast cancer biology, influencing tumour cell survival, metastasis, and resistance to therapy, particularly in triple-negative breast cancer, where traditional hormone receptors are absent. Together, these three targets provide complementary mechanisms of action—hormonal signalling (ER-α), cell cycle control [29], and stress response pathways – enabling identification of multitarget inhibitors or synergistic therapeutic combinations with broad applicability across different breast cancer molecular subtypes and potential to overcome therapeutic resistance [30,43].

The retrieved crystal structures underwent systematic preparation using the Protein Preparation Wizard in Schrödinger Suite to ensure optimal quality for molecular docking studies. Initially, all heteroatoms, including solvents, crystallographic waters, and metal ions, were removed from the structures, retaining only the protein and co-crystallised ligand to define the active site for subsequent docking procedures [44–46]. The bond orders were assigned, and appropriate ionisation states were generated for heteroatom groups. Hydrogen atoms were added to the structures to satisfy valency requirements, with proper orientation of hydroxyl groups, amide groups of asparagine and glutamine residues, and imidazole ring of histidine residues. Missing side chains and loops were modelled using Prime to ensure structural completeness. All disulfide bonds were created between proximal cysteine residues where appropriate. Finally, in the refine tab, sample water orientations with crystal symmetry are kept for the PROPKA at pH 7 to keep everything neutral [47]. Water molecules located beyond 3Å from the heteroatom groups were deleted to reduce computational complexity while retaining structurally important water molecules involved in ligand binding. While minimising each refined protein structure, we used the OPLS4 forcefield [36].

### 2.2. Pre-enumeration molecular docking

The complete prepared ligand library and generated grid file for each protein were individually selected for molecular docking with a virtual screening workflow (VSW) with all three docking scoring functions, namely HTVS, SP, XP, followed by MM-GBSA analysis [41,48–50]. Only the top 50% of data from HTVS was sent to the SP, 50% of SP was sent to the XP, and 100% to the MM-GBSA analysis. Further, after completing the analysis, we exported all the data to CSV format for filtering and analysis. In the Excel-based analysis (CSV files), we removed the ligand that shows a docking score greater than −5 (−4, −3, etc.) kcal/mol, and then we analysed the ligand that shows the binding affinity to the maximum proteins. After filtering, we found 15 drugs showing the best performance, with most of the proteins taken to the next level of the analysis.

### 2.3. Molecular drug enumeration and library preparation

The data generated from pre-enumeration molecular docking was further filtered, and finally, only the top fifteen drugs were exported in a separate project and further prepared for use in molecular enumeration. An automated reaction-based enumeration (pathfinder) tool was used to enumerate the ligands, the product similarity filter was kept to the top 10% of our compounds, and the number of maximum pathways was kept to 100 to produce a maximum of 10,000 compounds at max [35]. Further in the property filter, we kept the MolLogP (−1.50 to 5.50), MolWt (150–575), TPSA (30–150), Number of Chiral Centers (0–3), Number of H accepter (0–12), Number of H donor (0–5), MolMr (0–200), number of Rotatable bonds (0–10), heavy atoms count (0–100) and ring count (0–10) and validated the settings with the literature to get a well-optimised compound that can be fitted as a novel drug candidate and satisfy all criteria. Additionally, we checked the ADMET criteria of all compounds to filter the best compounds [51]. We again prepared the ligand library using LigPrep to fix the atomic issues, fulfil the missing bond angles, and generate stereoisomers and 3-D conformers, which generated 14750 compounds [34,35].

### 2.4. Post-enumeration docking and interaction fingerprints

The molecular enumeration generated a vast library, and the ligprep generated various stereoisomers and conformers, making the library much bigger than the original compound library. So, to reduce the computational cost, we applied the same HTVS, SP, and XP docking filter with strict parameters. In this scenario, we kept the filter for HTVS with a 10% pass, SP with a 10% pass, and XP with a 100% pass to the MM-GBSA analysis [35,41,52]. Further, we exported the data into CSV and performed all analyses in the Excel sheets. The same filtering criteria were used to keep the data showing a docking score less than −5 kcal/mol, and the rest of the data were omitted from the calculation sheet. After removal, we calculated the common drug candidates, which showed the best results in all cases. We filtered and took the top-performing drugs to redock using only the XP sampling algorithm to verify the results further. The interaction fingerprints of the docked complexes were computed with the Interaction Fingerprint tool in Maestro, in which we selected the option for Receptor-Ligand complexes, for any contacts, including backbone, side chain interaction, or any other possible interaction, and then aligned the sequences [35].

### 2.5. Filtering and Redocking

Due to the small library size, we have only taken the XP algorithm for the molecular docking that provides precise results with extensive sampling; however, it takes more time than other algorithms, and the same output files were taken for the MM-GBSA analysis [35,41,49]. The precise data was again exported to the CSV file for further analysis. The filtered data were analysed on behalf of the average, and the total docking score, the most complex, was fit, but the individual scoring and binding potential were manually re-analysed in Schrödinger’s Maestro workspace and taken as the top novel drug candidate for the other studies [35].

### 2.6. Lead optimisation (DFT, HOMO-LUMO analysis)

The DFT calculations for the opted compound *DdpMPyPEPhU* were performed with the Jaguar program in Schrödinger Maestro [35,53,54]. B3LYP-D3 was chosen as the significant default theory with an essential 6-31G** set in the input section. The DFT calculations, including Highest Occupied Molecular Orbital (HOMO) and Lowest Unoccupied Molecular Orbital (LUMO) energy gaps, chemical potential, and global reactivity descriptors, were used to evaluate the electronic stability and reactivity of the ligands. Compounds with lower HOMO–LUMO gaps and favourable reactivity indices were prioritised as they are more likely to interact effectively with the target proteins, guiding the selection of the most promising candidates for further docking and simulation studies. DFT was taken with SCF spin automatic treatment, optimised first excited state or time-dependent density functional theory (TDDFT), and used 3-body dispersion correction with all applicable dispersion-correlated functionals [55]. At the same time, the Hamiltonian was selected as nonrelativistic. Further, the accuracy level was kept as accurate instead of quick, with an atomic overlap in the initial guess. The convergence criteria were kept as maximum interaction of 48, energy change of 5e-05 Hartree, and root mean square (RMS) density matrix change of 5e-06, and in the convergence methods, self-consistent field (SCF) level shift was kept at 0.0 Hartree with thermal smearing of none by keeping the direct inversion of the iterative subspace (DIIS) convergence scheme [52].

In contrast, the orbitals were kept using consistent orbital sets when input structures were isomers, and all input structures used the same basic set with no final localisation. The 100 maximum steps were kept in the optimisation panel, with the switch to analytic integrals near convergence with default criteria. Schlegel guesses the initial Hessian with redundant internal coordinates and saves intermediate geometries to an output structures file to avoid losing them in between. In the properties panel, many properties were selected. However, the surfaces (molecular orbitals, density, potential) were the main ones that used the surface of electrostatic potential average local ionisation energy, with a box size of adjustment of 0.0 Å/side and grid density of 5.0 pts/Å respectively, while keeping the Kcal/mol as energy calculations to make it easier to understand. The non-covalent interactions were kept at a non-covalent grid density of 20.0 pts/Å, while the electron density was calculated among all densities along with the spin density. Moreover, Molecular orbitals for Alpha HOMO^−^ calculation were from 0 to LUMO^+^ 0 with the total number of orbitals two, while for the Beta, it was also kept identical for the HOMO^−^ and LUMO^+^ with NTO for excited states or TDDFT. In the solvation tab, the PBF solvent model was kept in the solvent media of water with an optimised gas-phase structure, and the output was kept in various formats to analyse it better after each job individually [35,54].

### 2.7. WaterMap analysis

WaterMap computation is an essential step in drug discovery for evaluating water molecule contributions within protein binding sites, which is essential for optimising drug-target interactions. This approach distinguishes between energetically unstable water molecules that can be advantageously displaced to improve binding affinity and thermodynamically stable waters that should be preserved. Through comprehensive analysis of hydration patterns and their influence on binding free energy, WaterMap facilitates lead optimisation and develops novel ligands. The method elucidates both entropic and enthalpic contributions of water molecules, thereby advancing structure-based drug design strategies and developing more potent and selective therapeutic compounds [ 56,57]. The computational workflow employed WaterMap’s Perform Calculation module, which utilises the Desmond molecular dynamics engine (D. E. Shaw Research - https://www.deshawresearch.com/) as its computational backend to determine water thermodynamics. The analysis focused on the ligand binding site derived from the docked conformation in the workspace, with the ligand retained throughout the calculation to ensure accurate analysis [ 56,58,59]. Water molecules within a 10 Å radius were examined using a 5-nanosecond MD simulation employing the OPLS4 force field, with water treated as explicit solvent. Trajectory files were not retained upon job completion to optimise computational efficiency. Subsequently, the WaterMap Perform Calculation interface was utilised to interpret results, with visualisation of receptors, ligands, hydrogen bonds, and energy markers to comprehensively assess the energetic contributions of water molecules in drug binding [ 58,59].

### 2.8. Molecular dynamics simulation and binding free energy calculation

MD simulation studies consume huge computational costs due to their algorithmic and loop complexity. Therefore, it provides enough evidence to rely on conducting in vitro and other experimental investigations. We used the Desmond package available in Schrödinger’s Maestro, developed by DE Shaw Research (https://www.deshawresearch.com/), which is the fastest and most accurate calculation among all MD algorithmic calculations [35,60,61]. A system-building tool was used to prepare the system using the SPC water model with an orthorhombic boundary condition of 10 x 10 Å spacing in buffer [62]. Partial atomic charges for all ligands were assigned automatically during ligand preparation using LigPrep under the OPLS4 force field. These force-field–consistent charges were retained during system building and molecular dynamics simulations. After excluding the ion and salt position within 20Å and adding the ion position, neutralise and minimise the system to build the whole system for MD simulation. In this study, we maintained the neutral charges by adding 1DI8; 4Cl^−^, 1NHZ; 6Na^+^, 3ERT; 6Na^+^ to each protein-ligand complex and applied the OPLS4 forcefield individually [36]. The system builder generated huge atoms after preparing the files in a water medium, and these are: 1DI8; 36,421, 1NHZ; 33,953, 3ERT; 32,998, respectively. Further, all building complexes were simultaneously simulated for 250 ns, with a recording interval (ps) at 250, with approximately 1000 frames for each in the NPT ensemble class [63]. The pressure was at 1.01325 bar at 300K temperature, and the model was relaxed before simulating it. Additionally, the simulated results were analysed using the simulation interaction diagram tool, and the protein-ligand deviation and fluctuation, ligand interaction, and its count at each nanosecond in the SPC water model were studied [35].

To further refine the computational results and evaluate the thermodynamically favourable ligand binding, we performed binding free energy calculations using the MM-GBSA approach as implemented in the Schrödinger Prime module. To account for dynamic effects, MM-GBSA calculations were carried out on multiple snapshots extracted at regular intervals from the equilibrated portion of the molecular dynamics trajectories. For each protein–ligand complex, representative frames were selected, and the binding free energy (ΔG_bind) was computed for each frame. The resulting per-frame binding energies were used to evaluate the binding energy distribution and temporal stability of ligand binding throughout the simulation. The average binding free energy and standard deviation were calculated from all sampled frames to provide a statistically robust estimate of binding affinity. This per-frame MM-GBSA analysis enables assessment of binding consistency and avoids bias associated with single-structure energy evaluation.

ΔG_bind = ΔE_MM + ΔG_solv − TΔS, where ΔE_MM includes van der Waals and electrostatic terms, and ΔG_solv includes polar and nonpolar solvation energies.

The calculations were carried out using Schrödinger’s thermal_mmgbsa.py script via the command line:

$SCHRODINGER/run thermal_mmgbsa.py JOB_NAME-out.cms

Binding energy values were collected across 1,000 frames from each 250 ns MD simulation. The total binding free energy was then divided into individual contributions, including van der Waals forces, electrostatic interactions, polar solvation, and nonpolar solvation components. These values allowed us to quantitatively compare the binding strength of different ligand–target complexes and helped identify the most promising candidate.

## 3. Results

### 3.1. Data refinement and pre-enumeration docking

We prepared the final list of 316 FDA-approved drugs against breast cancer using LigPrep, which generated 897 stereoisomers. The same library of 897 compounds was used for molecular docking studies with 3 proteins. The data was exported to CSV for analysis, which kept only ligands showing a docking score below −5 Kcal/mol and deleted all ligands whose docking score was more than −5 Kcal/mol. The top 15 drugs showed the best result in the case of all three proteins, and then we validated them with all ADMET criteria kept for the subsequent analysis.

### 3.2. Drug enumeration for automated drug redesigning

For the molecular enumeration, we used the ‘Automated Reaction-Based Enumeration’ tool that generated 14,750 novel drug candidates from the 15 drug backbones resulting from pre-enumeration docking. The generated novel candidates were in raw form, and to convert them into the 3-D form, they needed to be prepared again to satisfy the angles and bonding configuration, resulting in 84,941 stereoisomers. All parameters HTVS, SP, and XP were set to 10% for the docking set and passed to the next level to impose a lower computational cost, followed by MM-GBSA calculation. Nevertheless, we found very ambiguous and unsatisfactory data, as only a few drugs showed docked positions in a better configuration. We again exported the top result of 967 compounds out of 84,941, as it has shown the docking scores less than −5 Kcal/mol.

### 3.3. Post-enumeration docking and interaction analysis

**Fig 2:**
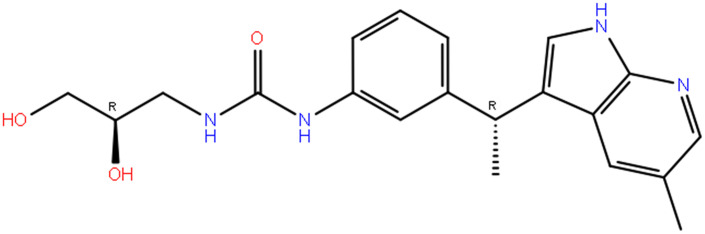
Molecular structure of ligand DdpMPyPEPhU against Breast Cancer.

At this stage, we had a short library of 967 compounds, and we did only XP docking using ligand docking and export data in CSV, after filtering and analysing data, we found novel drug candidates which is unique and whose average and total docking scores were best, after searching, filtering, we found DdpMPyPEPhU as a lead like compound that was validated against breast cancer with advanced analysis. The structure of the enumerated novel compound is shown in Fig 2. The ligand interaction diagram analyses for each complex were filled with good binding potentials with Hydrogen bonds, pi-pi interactions, and pi-cations*.* The DdpMPyPEPhU with the CDK2 (PDB ID: 1DI8) complex has shown a docking score of −11.424 Kcal/mol and MM-GBSA score of −39.01 Kcal/mol (Table 1) while interacting with many hydrogen bonds among the ASP86 residue with the OH atom; GLU81, and ILE10 residues is interacting with the NH atom, and LEU83 is interacting with the N atom of the ligand. Additionally, TYR142 and LYS33 residues also form a pi-cation interaction with the benzene ring. The GR (PDB ID: 1NHZ) with DdpMPyPEPhU complex shows.

**Table 1:** Comparative analysis of the Binding affinity, MM-GBSA, and RMSD with other essential computational scores of DdpMPyPEPhU and control FDA drugs Lapatinib and Tamoxifen against the CDK2 (PDB ID: 1DI8), ER-α (PDB ID: 3ERT), and GR (PDB ID: 1NHZ) receptors.

Compounds	DdpMPyPEPhU (Enumerated)			Lapatinib (Control 1)			Tamoxifen (Control 2)		
Receptors	**1DI8**	**1NHZ**	**3ERT**	**1DI8**	**1NHZ**	**3ERT**	**1DI8**	**1NHZ**	**3ERT**
Resolution	2.2 Å	2.3 Å	1.9 Å	2.2 Å	2.3 Å	1.9 Å	2.2 Å	2.3 Å	1.9 Å
*Docking Score*	−11.424	−11.239	−10.987	−6.744	−8.798	−8.067	−5.258	−7.092	−9.034
*MM-GBSA*	−39.01	−56.52	−47.01	−69.53	−82.34	−83.37	−59.18	76.8	−86.3
*Prime Hbond*	−154.89	−140.58	137.66	−277.95	−175.11	−168.15	−277.84	−174.66	−166.46
*Prime VdW*	−1397.51	−928.01	−1078.04	−1859.51	−1030.84	−1088.32	−1869.35	−1040.37	−1092.04
*RMSD*	1.07Å	1.22Å	1.04Å	1.4Å	1.4Å	1.32Å	2.04Å	1.96Å	1.57Å

**Fig 3:**
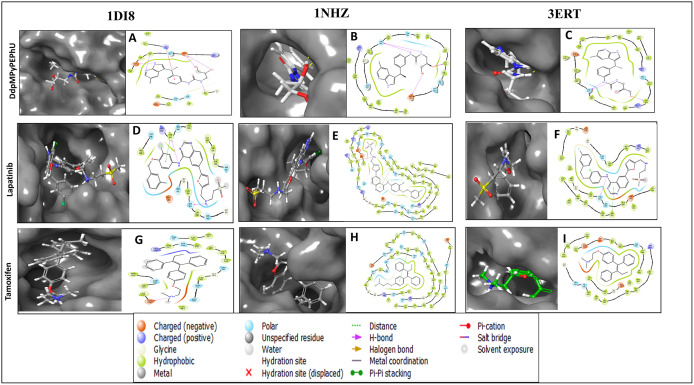
Docked complexes along with the 3D surface view and 2D ligand interaction diagram of DdpMPyPEPhU and control FDA drugs Lapatinib and Tamoxifen with the CDK2 (PDB ID: 1DI8), B) GR (PDB ID: 1NHZ), and C) ER-α (PDB ID: 3ERT) receptors.

Favourable score of −11.239 Kcal/mol and MM-GBSA score of −56.52 Kcal/mol (Table 1) while interacting with a hydrogen bond with ASP742, and CYS736 residues with OH atoms, and ASN564 residue interacts with the NH atom (Fig 3). DdpMPyPEPhU interaction with ER-α (PDB ID: 3ERT) has shown a docking score of −10.987 Kcal/mol and MM-GBSA score of −47.01 Kcal/mol while interacting with a hydrogen bond MET528 residue with OH atoms, and THR347 residue is interacting with two NH atoms of the ligand. Lapatinib shows stronger and more stable interactions with all three receptors than tamoxifen. Its binding affinities (−6.744 to −8.798 kcal/mol) are consistently better, especially with the glucocorticoid and estrogen receptors, while its MM-GBSA values (−69.53 to −83.37 kcal/mol) indicate greater overall complex stability. Lapatinib also forms more hydrogen bonds and stronger van der Waals interactions, which is reflected in its lower energy values, and its Root Mean Square Deviation (RMSD) range (1.32–1.4 Å) suggests the ligand fits well within the binding sites with minimal structural fluctuations. On the other hand, tamoxifen binds less strongly (−5.258 to −9.034 kcal/mol) and shows slightly higher RMSD values (up to 2.04 Å), indicating less stable docking poses, particularly with CDK2 and the glucocorticoid receptor. Fig 3 displays the docked complexes – CDK2 (PDB ID: 1DI8), B) GR (PDB ID: 1NHZ), and C) ER-α (PDB ID: 3ERT) receptors with ligand DdpMPyPEPhU and FDA drug controls – lapatinib and tamoxifen, along with the interactions formed between each of them.

A quantitative residue-level interaction analysis was performed to evaluate the multitarget binding profile of DdpMPyPEPhU across ER-α, GR, and CDK2. The comparison revealed that approximately 40–45% of the interacting residues were conserved among all three targets, representing shared anchoring interactions responsible for stable binding. The remaining interactions were target-specific, reflecting structural complementarity within each binding pocket. This combination of conserved and unique interactions supports the true multitarget nature of DdpMPyPEPhU, enabling simultaneous engagement of hormone signaling and cell-cycle regulatory proteins rather than nonspecific binding.

### 3.4. Molecular interaction fingerprints (MIFs) and pharmacokinetics studies with QikProp and ProTox-3.0 platform

**Fig 4:**
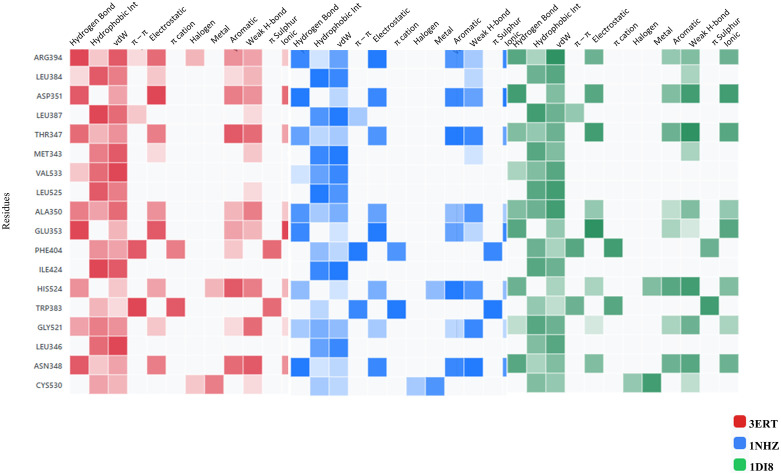
The molecular interaction fingerprints of docked poses of CDK2 (PDB ID: 1DI8), B) ER-α (PDB ID: 3ERT), C) GR (PDB ID: 1NHZ) receptors with DdpMPyPEPhU.

The MIFs represent the spatial arrangement of molecular interactions between a ligand and its target protein. MIFs help interpret docked complexes in multitargeted drug design by providing insight into the specific interactions occurring at the binding site. By analysing MIFs, the common interaction patterns across multiple targets facilitate the design of compounds with enhanced polypharmacological profiles. This approach enables the rational design of drugs that simultaneously modulate multiple targets, improving therapeutic efficacy and reducing side effects. The fingerprints of all complexes have revealed the most interacting residues with counts of ARG394, LEU384, ASP351, LEU387, THR347, MET343, VAL533, LEU525, ALA350, GLU353, PHE404, ILE242, HIS524, TRP383, GLY521, LEU346, ASN348, and CYS530. Fig 4 displays the combined heatmap plots representing the molecular interaction fingerprints of all three receptors docked with the ligand DdpMPyPEPhU. The counts of interaction from fingerprints for each residue type in the interaction fingerprints can help infer how different residue types contribute to the interactions with the drug candidate DdpMPyPEPhU. The hydrophobic residues, such as LEU, VAL, ALA, ILE, MET, PHE, and TYR, are likely involved in interactions between nonpolar molecules or parts of molecules in aqueous environments, where hydrophobic residues of the protein interact with hydrophobic regions of the drug compound. The high hydrophobic residue count suggests that DdpMPyPEPhU may have significant hydrophobic characteristics. The polar residues, such as THR, GLU, ASN, HIS, ASP, ARG, and LYS, typically participate in polar interactions such as hydrogen bonding, electrostatic interactions, and salt bridges and involve the attraction between polar or charged groups in the protein and the drug compound. Polar residues in the interaction fingerprints indicate potential hydrogen bonding or electrostatic interactions between the drug compound and the protein. CYS may contribute to interactions through unique structural features or specific interactions such as pi-stacking (aromatic residues like TRP and TYR), disulfide bonding (CYS) or specialised interactions involving histidine tautomers (Fig 4). CYS lower counts suggest it may play a lesser role than hydrophobic and polar residues. The distribution of residue types in the interaction fingerprints suggests that DdpMPyPEPhU likely interacts with the protein through a combination of hydrogen, hydrophobic, ionic, and other types of interactions. This information can guide further investigations into the specific binding mode and efficacy of DdpMPyPEPhU as a drug compound.

Further, the ADMET properties of the compound DdpMPyPEPhU are completely satisfactory for all the QikProp parameters used for the computations, providing valuable insights into its pharmacokinetic and pharmacodynamic characteristics [64,65]. Notably, it exhibits a QPlogPo/w value of 1.555, indicating moderate lipophilicity within the acceptable range (Table 2). Additionally, it demonstrates a negative QPlogS value of −3.904, implying low aqueous solubility, which might necessitate formulation strategies for optimal delivery. Despite this, DdpMPyPEPhU boasts favourable predictions for human oral absorption (69.84%), suggesting good bioavailability. Regarding its potential to cross the blood-brain barrier (BBB), the compound presents a QPlogBB value of −2.073, suggesting limited brain penetration, which could be advantageous in avoiding CNS side effects or targeting peripheral receptors. Importantly, it shows no significant concerns for hERG inhibition (QPlogHERG: −4.399), indicating minimal risk of arrhythmia. Moreover, the compound exhibits desirable physicochemical properties, with a MW of 368.435 g/mol falling within the acceptable range. It possesses eight rotatable bonds, suggesting sufficient flexibility for binding interactions without excessive conformational complexity. The compound’s dipole moment of 8.891 D indicates polarity, which could influence its interactions with biological targets. Metabolism predictions suggest moderate metabolic stability, with DdpMPyPEPhU potentially undergoing seven metabolic transformations. It demonstrates a favourable prediction for Caco-2 cell permeability (QPlogPCaco: 77.224), indicating good intestinal absorption (Table 2). Furthermore, the compound’s predicted volume (1188.122 Å³) falls within the acceptable range, suggesting an adequate size for efficient binding to biological targets. The ADMET profile of DdpMPyPEPhU suggests promising pharmacokinetic properties, including favourable oral absorption, metabolic stability, and potential for intestinal permeability. While its aqueous solubility and BBB permeability may pose challenges, it is safe for all seven cancers (Table 2). These insights provide a foundation for further preclinical evaluation and optimisation of DdpMPyPEPhU as a potential drug candidate.

**Table 2:** Comparative ADMET representing the computational prediction of DdpMPyPEPhU with QikProp.

QikProp Descriptors	Standard Values	DdpMPyPEPhU	Lapatinib	Tamoxifen
Formula	–	C20H24N4O3	C29H26ClFN4O4S	C26H29NO
QPlogPo/w	−2.0–6.5	1.555	5.87	6.84
#stars	0–5	0	3	1
QPlogS	−6.5–0.5	−3.904	−7.12	−7.58
#amine	0–10	2	1	–
CIQPlogS	−6.5–0.5	−4.11	−7.38	−7.82
#amidine	0	0	0	–
QPlogHERG	concern below −5	−4.399	−5.12	−5.34
#acid	0–10	1	0	–
QPPCaco	<25 poor,>500 great	77.224	124.5	1842.5
#amide	0–1	2	0	–
QPlogBB	−3.0–1.2	−2.073	−1.28	−0.24
#rotor	0–15	8	11	–
QPPMDCK	<25 poor,>500 great	44.893	68.3	986.4
#rtvFG	0–2	1	0	–
QPlogKp	−8.0 – −1.0	−3.822	−3.94	−2.84
CNS	−2 (inactive), + 2 (active)	−2	−1	0
IP (eV)	7.9–10.5	8.489	8.24	7.96
mol MW	130.0–725.0	368.435	581.058	371.514
EA (eV)	−0.9–1.7	0.244	0.68	0.14
#metab	1–8	7	7	6
dipole	1.0–12.5	8.891	9.42	3.87
SASA	300.0–1000.0	678.373	897.24	748.62
QPlogKhsa	−1.5–1.5	−0.315	1.18	1.42
FOSA	0.0–750.0	261.95	412.6	524.8
Human Oral Absorption	N/A	2	2	3
FISA	7.0–330.0	206.695	268.4	198.6
% Human Oral Absorption	>80% is high, < 25% is poor	69.84	32.4	67.8
PISA	0.0–450.0	209.729	216.2	25.2
SAfluorine	0.0–100.0	0	22.8	0
WPSA	0.0–175.0	0	0	0
SAamideO	0.0–35.0	18.6	31.4	0
volume	500.0–2000.0	1188.122	1687.5	1206.8
PSA	7.0–200.0	117.753	106.35	12.47
donorHB	0.0–6.0	5	2	0
#NandO	2–15	7	9	2
accptHB	2.0–20.0	6.4	7.5	1.5
Rule Of Five	maximum is 4	0	2	1
dip²/V	0.0–0.13	0.066533	0.0526	0.0124
Rule Of Three	maximum is 3	5	8	6
ACxDN^.5/SA	0.0–0.05	0.0210958	0.0328	0.0089
#ringatoms	N/A	0	24	18
glob	0.75–0.95	0.799718	0.823	0.764
#in34	N/A	1	5	2
QPpolrz	13.0–70.0	38.261	62.4	48.6
#in56	N/A	0	5	0
QPlogPC16	4.0–18.0	13.346	15.87	17.24
#noncon	N/A	27	35	28
QPlogPoct	8.0–35.0	24.315	28.34	32.87
#nonHatm	N/A	13	9	28
QPlogPw	4.0–45.0	17.423	31.62	35.41
Jm	N/A	0.007	0.0186	–

Further, to evaluate the safety profile of DdpMPyPEPhU in the context of drug repurposing, machine-learning–based toxicity predictions were performed using the ProTox-3.0 platform. ProTox-3.0 integrates deep neural network models trained on experimentally validated toxicological datasets to classify compounds into toxicity classes and to estimate their LD₅₀ values. Predictions were obtained across major toxicity endpoints, including organ toxicity, mutagenicity, carcinogenicity, cytotoxicity, immunotoxicity, and blood–brain barrier (BBB) permeability, along with nuclear receptor signaling, stress-response pathways, cytochrome P450 interactions, and molecular initiating events (MIEs). The compound was predicted to be non-hepatotoxic (DILI inactive, probability = 0.76) and non-cardiotoxic (inactive, probability = 0.70), indicating a favorable safety profile for critical organs. However, neurotoxicity (active, 0.74), nephrotoxicity (active, 0.61), and respiratory toxicity (active, 0.88) were predicted, suggesting potential liabilities that warrant careful experimental validation (Supplementary Sheet ProTox-3.0).

### 3.5. DFT analysis

**Fig 5:**
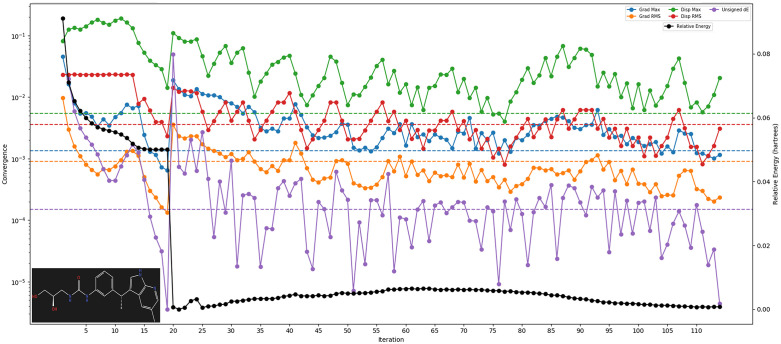
The DFT-based energy of DdpMPyPEPhU during 115 optimisation steps against relative energy (hartrees). The black colour shows relative energy, the blue colour shows Grad maximum, the green colour shows displacement maximum, the Violet colour shows unsigned dE, the orange colour shows Grad RMS, and the red colour shows displacement RMS.

The DFT is a quantum mechanics simulation method to estimate various properties of molecules, crystals, atoms, surfaces, and drug candidates. The optimisation of the drug candidate DdpMPyPEPhU compound shows solid stability. The HOMO and the LUMO were at, respectively, while the lowest single excitation and the singlet oscillator strength were. Further, the relative energy (hartrees), Grad maximum, displacement maximum, unsigned dE, Grad RMS, and displacement RMS are shown in Fig 5 with different colours against the iteration count. The iteration count was 110 to reach the global minima, which performed almost constantly after 20 iterations. The DFT results have provided a comprehensive insight into its electronic structure, energetics, and vibrational properties. Utilising the B3LYP-D3 functional and 6-31g** basis set, the calculations yielded a gas phase energy of −1221.373659 Hartrees. The absence of solvation energy indicates the examination of the molecule in its isolated state. Analysis of molecular orbitals reveals a HOMO energy of −0.751631 Hartrees and a LUMO energy of −0.602109 Hartrees, indicative of its electronic structure and potential reactivity. Furthermore, the compound exhibits a dipole moment of 9.2975 Debye, with components along the X, Y, and Z axes, suggesting polarity and asymmetry in charge distribution. Vibrational analysis reveals the molecule’s lowest and highest frequencies at 22.050979 cm^ − 1 and 3741.607344 cm^ − 1, respectively, indicating its characteristic vibrational modes and potential for infrared absorption. Notably, the absence of negative frequencies indicates the molecule’s stability. Thermodynamic properties such as entropy, enthalpy, and free energy provide crucial insights into the compound’s stability and reactivity under standard conditions. The calculated values at 298.15K and 1.00 atm include an entropy of 161.610462 Kcal/mol, an enthalpy of 15.855055 kcal/mol, and a free energy of −32.329104 kcal/mol, suggesting favourable thermodynamic stability (Fig 5). The insight into DFT computations for the compound DdpMPyPEPhU provides molecular properties, potential reactivity, and stability. The calculated gas phase energy reflects the stability of the compound in isolation, with a negative value indicating a favourable energy state. The absence of solvation energy suggests that the calculations were conducted without considering any surrounding solvent molecules, which is common for initial electronic structure studies. The analysis of molecular orbitals, specifically the HOMO and LUMO energies, offers valuable information regarding the compound’s electronic structure and reactivity. The HOMO–LUMO energy gap of DdpMPyPEPhU reflects its electronic stability and chemical reactivity, which directly influence its ability to form and maintain noncovalent interactions with protein residues. Regions of high electron density identified by the molecular electrostatic potential map correspond to hydrogen bond donor and acceptor sites observed in docking and MD simulations, while π-electron-rich regions facilitate π–π stacking with aromatic residues in ER-α, GR, and CDK2. These electronic features explain the strong and persistent binding of DdpMPyPEPhU to all three targets, thereby linking the DFT descriptors to its predicted biological activity.

### 3.6. WaterMap computation

The WaterMap calculation identified 113 distinct hydration sites within the binding pocket of the 1DI8 protein structure, revealing significant opportunities for structure-based drug optimisation. The analysis revealed a diverse energetic landscape with occupancy values ranging from 0.28 to 1.00, where 19 high-occupancy sites (≥0.80) represent the most stable water positions and 50 low-occupancy sites (<0.50) indicate more transient hydration. Thermodynamically, several sites present excellent displacement opportunities, particularly Site 24 (ΔG = +8.54 kcal/mol), Site 6 (ΔG = +6.67 kcal/mol), and Site 12 (ΔG = +6.71 kcal/mol), which are highly unfavourable and represent prime targets for hydrophobic ligand substitution that could significantly enhance binding affinity. Conversely, energetically favourable sites such as Site 88 (ΔG = −1.71 kcal/mol), Site 50 (ΔG = −1.59 kcal/mol), and Site 42 (ΔG = −1.36 kcal/mol) should be preserved in drug design as they likely mediate important stabilising interactions. The hydrogen bonding analysis revealed an average of 1.67 water-water interactions and 0.95 protein-water interactions per site, with no ligand-water interactions observed (all #HB(LW) = 0.00), indicating the ligand is not currently engaging with the water network. Overall, identifying multiple high-energy water sites (>2.0 kcal/mol) provides clear guidance for lead optimisation strategies, where displacing these unfavourable waters through appropriate chemical modifications could yield substantial improvements in binding affinity and selectivity.

The watermap analysis of the GR (PDB ID:1NHZ) protein receptor showed similar properties. In complex with the ligand, 113 water binding sites were identified with diverse thermodynamic and structural properties. Site occupancy values ranged from 0.28 to 1.00, with only 10 sites (8.8%) showing high occupancy (≥0.90), 58 sites (51.3%) exhibiting medium occupancy (0.50–0.89), and 45 sites (39.8%) displaying low occupancy (<0.50). The thermodynamic analysis revealed that only 23 sites (20.4%) showed favourable binding free energies (dG < 0), with Site 50 being the most favourable at dG = −1.59 kcal/mol, while the majority of sites (79.6%) exhibited unfavourable binding energies, with Site 24 showing the most unfavourable binding at dG = 8.54 kcal/mol. Enthalpically, 48 sites (42.5%) showed favourable binding (dH < 0), with Site 1 displaying the most favourable enthalpy at dH = −5.63 kcal/mol, while entropic contributions were consistently unfavourable across all sites, ranging from 0.56 to 5.24 kcal/mol. The hydrogen bonding analysis demonstrated that water-water interactions averaged 1.67 bonds per water molecule (range: 0.02–2.77), protein-water interactions averaged 0.94 bonds per water molecule (range: 0.00–2.98), with 16 sites (14.2%) showing no protein-water hydrogen bonds, while ligand-water interactions were absent across all sites. Overlap analysis indicated that 17 sites (15.0%) showed overlap with other water binding locations, with overlap values ranging from 0.03 to 1.00, suggesting potential competition for binding space at these locations. The results indicate significant heterogeneity in water binding behaviour, with most sites representing thermodynamically unfavourable but kinetically accessible binding locations that may play important roles in protein dynamics and ligand binding processes.

**Fig 6:**
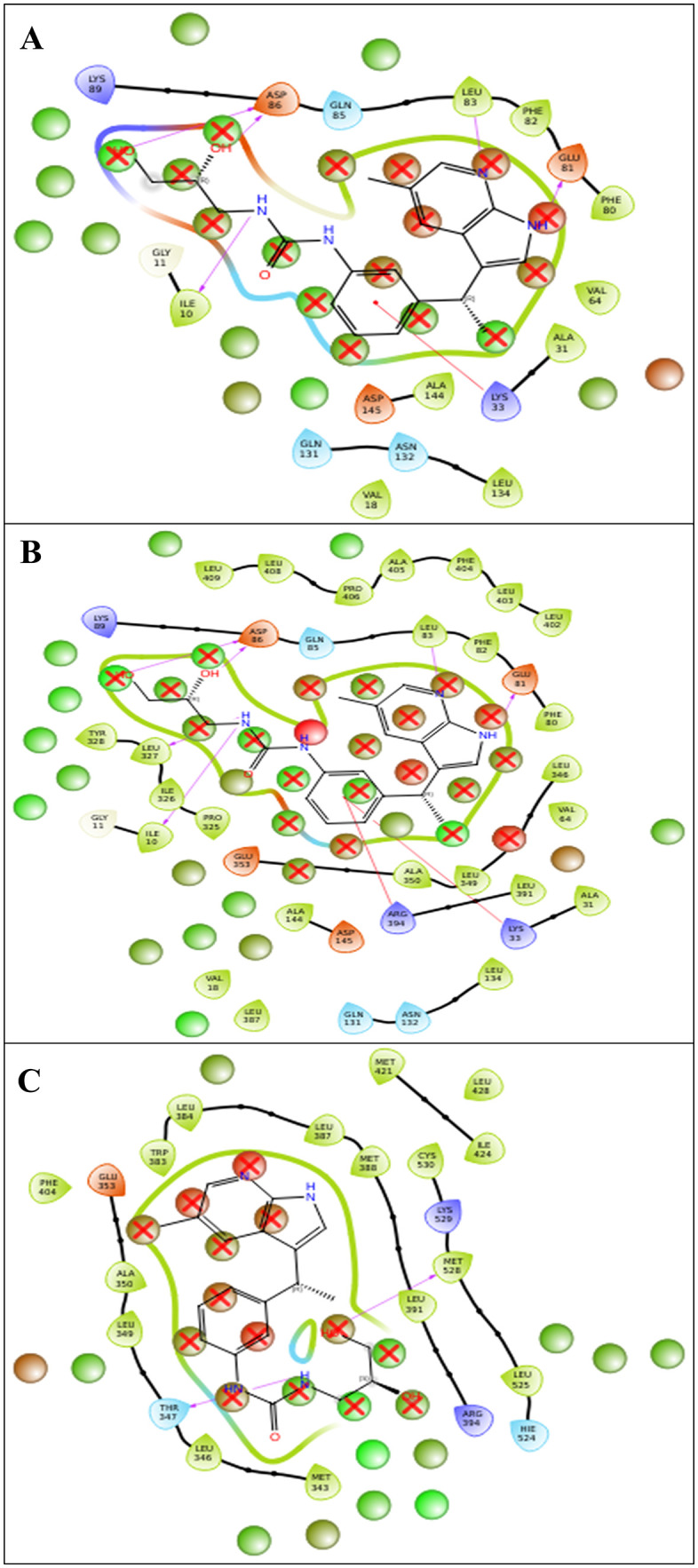
WaterMap analysis of the receptor-ligand complexes CDK2 (PDB ID: 1DI8), B) ER-α (PDB ID: 3ERT), C) GR (PDB ID: 1NHZ) receptors with DdpMPyPEPhU, revealing key hydration sites.

Similarly, the watermap calculation for the PDB ID: 3ERT protein structure identified 113 distinct hydration sites with occupancy values ranging from 0.28 to 1.00, revealing an identical binding site water distribution pattern to the previously analysed 1DI8 structure. The thermodynamic analysis identified several exceptionally unfavourable hydration sites that represent prime displacement targets, most notably Site 24 (ΔG = +8.54 kcal/mol), Site 6 (ΔG = +6.67 kcal/mol), and Site 12 (ΔG = +6.71 kcal/mol), which exhibit highly positive free energy values and would benefit significantly from hydrophobic ligand substitution to enhance binding affinity. Additional high-energy sites, including Site 5 (ΔG = +5.48 kcal/mol), Site 37 (ΔG = +4.69 kcal/mol), Site 112 (ΔG = +4.60 kcal/mol), Site 40 (ΔG = +4.28 kcal/mol), Site 13 (ΔG = +4.10 kcal/mol), and Site 110 (ΔG = +4.10 kcal/mol), also present excellent opportunities for medicinal chemistry optimisation through strategic displacement. Conversely, energetically favourable sites such as Site 88 (ΔG = −1.71 kcal/mol), Site 50 (ΔG = −1.59 kcal/mol), Site 71 (ΔG = −1.40 kcal/mol), Site 42 (ΔG = −1.36 kcal/mol), and Site 19 (ΔG = −1.29 kcal/mol) should be preserved in drug design as they likely mediate critical stabilising interactions with the protein. The hydrogen bonding network analysis revealed an average of 1.67 water-water interactions and 0.95 protein-water interactions per site, with no ligand-water interactions observed (all #HB(LW) = 0.00), indicating the current ligand does not engage with the hydration network. The identification of 19 high-occupancy sites (≥0.80) representing stable water positions and multiple high-energy displacement targets (>4.0 kcal/mol) provides clear structure-activity relationship guidance for lead optimisation, where strategic displacement of these thermodynamically unfavourable waters could yield substantial improvements in binding potency and selectivity. Fig 6 depicts the protein-ligand interaction in a water-simulated environment, showing interactions between the residues. The Figure illustrates the hydration thermodynamics around the ligand-binding pocket, as computed by Schrödinger’s WaterMap module. Red spheres represent high-energy hydration sites (unfavourable waters with ΔG > 0 kcal/mol), indicating regions where water displacement by ligand atoms may confer a binding advantage. Blue spheres denote favourable hydration sites (ΔG < 0 kcal/mol), typically involved in stabilising water-mediated interactions. The receptor surface is shown in a semi-transparent rendering, and the bound DdpMPyPEPhU is visualised in stick representation. This mapping helps identify potential hotspots for ligand optimisation through strategic water displacement. High-energy hydration sites located near polar and heteroaromatic regions of the binding pocket were displaced by DdpMPyPEPhU, indicating entropically favourable regions for ligand binding. These sites suggest that future chemical optimisation could introduce polar substituents or hydrogen-bond donors/acceptors at these positions to further enhance binding affinity through structured water replacement.

### 3.7. Molecular dynamics simulation

MD simulations are sophisticated algorithms that analyse the complex’s behavioural connections, determining the complex’s deviation, fluctuation, interaction, and other points using the generated trajectories. We analysed the trajectories generated after 250 ns in the simulation investigation and thoroughly investigated the deviation, fluctuation, and ligand interaction count unique to amino acids. MD simulations for the three complexes were performed using the Desmond package in the Schrödinger suite to illuminate the strength of identified compounds after molecular docking.

#### 3.7.1. Root mean square deviation (RMSD).

**Fig 7:**
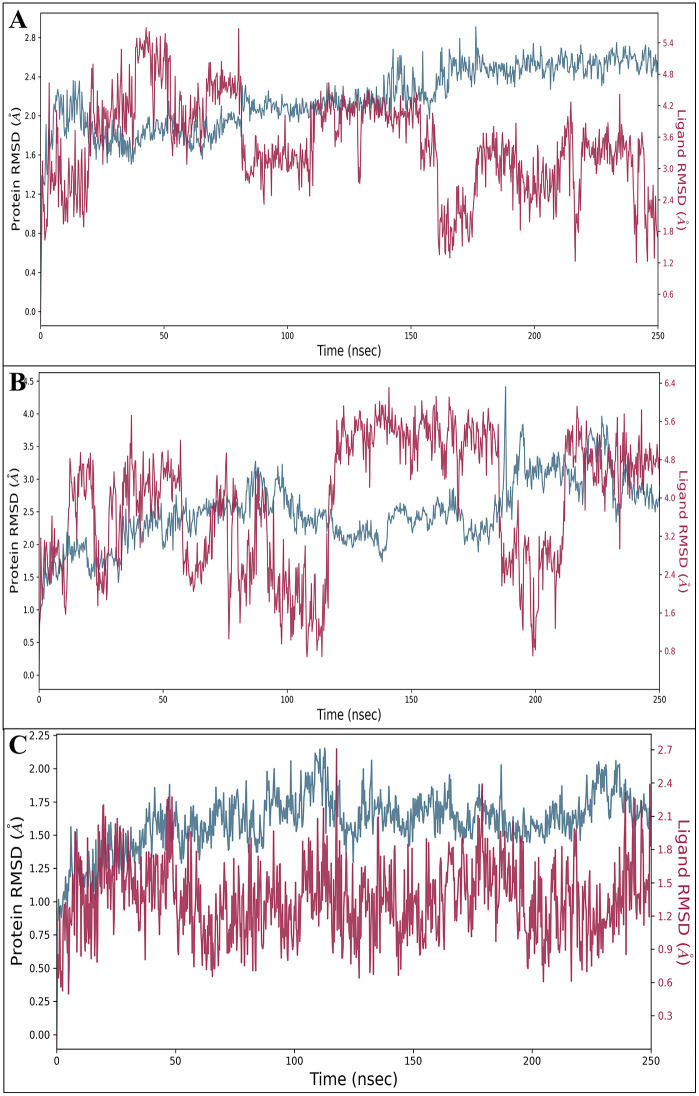
Post molecular dynamics simulation analysis; the Root Mean Square Deviation (RMSD) DdpMPyPEPhU in complex with A) CDK2 (PDB ID: 1DI8), B) ER-α (PDB ID: 3ERT), and C) GR (PDB ID: 1NHZ) receptors, respectively.

The RMSD values offer the deviation within the protein and ligand at the Å stage with time intervals in the ns stage, meaning the RMSD provides proof of consistent overall performance. The RMSD is the average displacement of the atoms at a simulation time point relative to a reference structure, usually the primary frame of the simulation or the crystallographic structure. The ER-α (PDB ID:3ERT) in complex with DdpMPyPEPhU initially deviated to 1.23Å while for the ligand to 1.10Å at 0.25 ns, and during the entire simulation period, it showed steady performance, and at 250 ns, the protein deviation was 2.54Å, and the ligand deviation was 1.72Å; after ignoring the initial phase, the RMSD of the complex is acceptable. The CDK2(PDB ID: 1DI8) in complex with DdpMPyPEPhU initially at 0.25 ns, the protein deviated at 0.92 Å and the ligand at 1.37Å, at 250 ns it produces a deviation of protein 2.57Å while the ligand 4.93Å. After ignoring the initial deviations, the performance shows that the ligand performed better. GR (PDB ID: 1NHZ) in complex with DdpMPyPEPhU initially deviated at 0.25 ns in the state of protein at 0.86Å and the ligand at 0.73Å. At 250 ns, the protein deviated at 1.68Å and for the ligand at 1.83Å; after ignoring the first ns, the complex performed the best stability. The consistent performance and stability observed in the RMSD graphs indicate that DdpMPyPEPhU forms stable complexes with the target proteins over time. This stability is essential for the compound to interact with the proteins and exert its therapeutic effects effectively. Furthermore, the RMSD values reveal that the initial deviations observed in some complexes are transient and often attributed to factors such as sudden changes in heat or alterations in the solute medium. However, these initial deviations do not compromise the overall stability of the complexes, as evidenced by the subsequent steady performance of DdpMPyPEPhU. Additionally, the RMSD analysis highlights that DdpMPyPEPhU demonstrates stable performance across proteins implicated in breast cancer. Moreover, the observed stability of DdpMPyPEPhU complexes after ignoring initial deviations underscores the compound’s reliability and potential as a therapeutic agent against cancer. This reliability is crucial for ensuring consistent efficacy and minimising the risk of resistance development in cancer treatment. Fig 7 displays the RMSD of DdpMPyPEPhU in complex with A) CDK2 (PDB ID: 1DI8), B) ER-α (PDB ID: 3ERT), and C) GR (PDB ID: 1NHZ) receptors, respectively.

#### 3.7.2. Root mean square fluctuation (RMSF).

**Fig 8:**
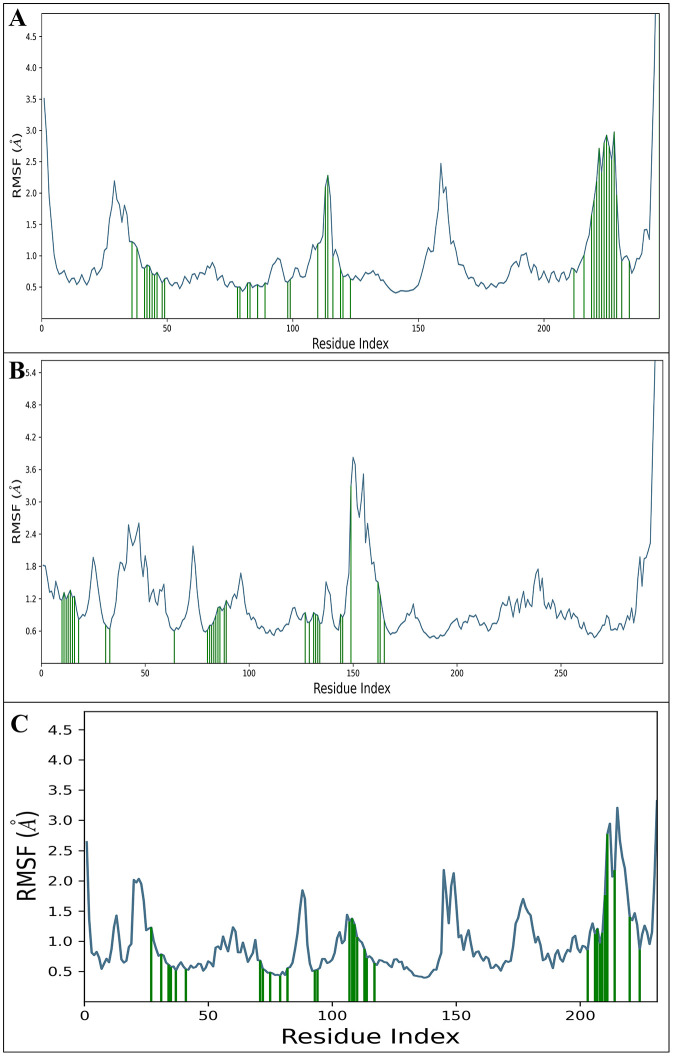
Post molecular dynamics simulation analysis; the Root Mean Square Fluctuation (RMSF) DdpMPyPEPhU in complex with A) CDK2 (PDB ID: 1DI8), B) ER-α (PDB ID: 3ERT), and C) GR (PDB ID: 1NHZ) receptors, respectively.

The RMSF gives the value of protein-ligand fluctuations at the residue and atomic levels. It is a proportion of the displacement of a specific molecule or gathering of ions compared with the reference structure, found by averaging over the number of particles. The DdpMPyPEPhU with residues of ER-α (PDB ID: 3ERT) are SER341, MET343, LEU346, THR347, ASN348, LEU349, ASP351, GLU353, LEU354, TRP383, LEU384, LEU387, MET388, LEU391, ARG394, LEU403, PHE404, GLY415, MET421, ILE424, PHE425, LEU428, MET517, GLY521, HIS524, LEU525, VAL534, PRO535, LEU536, and LEU539 and only a few residues fluctuated beyond 2Å are VAL418, GLU419, TYR526, SER527, MET528, LYS529, CYS530, LYS531, ASN532, and VAL533 (Fig 8). The DdpMPyPEPhU molecules interacted with of CDK2 (PDB ID:1DI8) residues are ILE10, GLY11, GLU12, GLY13, THR14, TYR15, GLY16, VAL18, ALA31, LYS33, VAL64, PHE80, GLU81, PHE82, LEU83, HIS84, GLN85, ASP86, LYS88, LYS89, ASP127, LYS129, GLN131, ASN132, LEU133, LEU134, ALA144, ASP145, GLU162, VAL163, and THR165, one residues fluctuated beyond 2Å that are ALA149. The DdpMPyPEPhU molecules with GR (PDB ID: 1NHZ) residues interacted with TRP556, MET560, THR561, LEU563, ASN564, LEU566, GLN570, TRP600, MET601, MET604, LEU608, ARG611, CYS622, PHE623, LEU636, PRO637, ASP638, MET639, GLN642, MET646, LEU732, TYR735, CYS736, PHE737, GLN738, and THR739, and many residues fluctuated beyond 2Å are PHE740, LYS743, PHE749 and LEU753. A detailed view is presented in Fig 8 for RMSF, and the observation of stable interactions between the compound and specific residues within proteins associated with these cancers suggests that DdpMPyPEPhU could effectively target and inhibit the activity of key proteins involved in cancer progression. Furthermore, the limited fluctuation of residues beyond 2Å in ER-α (3ERT) indicates robust and stable binding between DdpMPyPEPhU and the target protein. This stability is essential for ensuring sustained inhibition of cancer cell growth and proliferation. Moreover, the ability of DdpMPyPEPhU to form stable complexes with multiple proteins suggests its potential as a broad-spectrum anticancer agent.

#### 3.7.3. Simulative interaction analysis.

**Fig 9:**
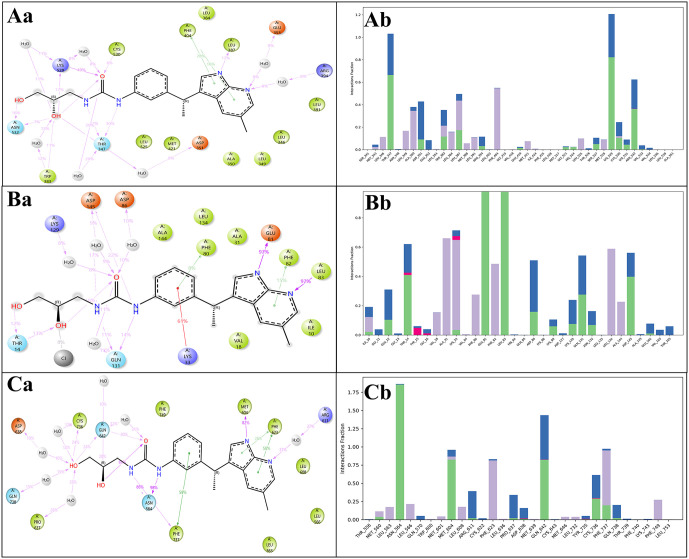
Simulative interactive analysis. (a) Simulative interaction diagram. (b) The bar graph for the interaction counts of DdpMPyPEPhU with A) CDK2 (PDB ID: 1DI8), B) Human Estrogen Receptor-α (PDB ID: 3ERT), and C) GR (PDB ID: 1NHZ), respectively.

The simulative interaction diagram (SID) shows the ligand contacts with the protein throughout the simulation periods and how the electron transfer occurs, providing a more in-silico promising ligand binding to grasp the position from a dynamic angle. The SID makes determining which amino acids interact with the ligand’s atoms easier during the simulation. Using interaction analysis and the simulated interactions diagram tool, we examined the 2-D interactions between the proteins and the DdpMPyPEPhU ligand at their binding sites. ER-α (PDB ID: 3ERT) in complex with DdpMPyPEPhU found that many water molecules are present for complex stability. Hydrogen bonds interact with different residues, whereas ASN532, TRP383, ASP351, and LYS529 residues along the OH atom; LYS529, CYS530 residues, and THR347 residue with water molecules interacting with the O atom; two NH atoms contact the THR347 residue; and two N atoms along ARG394, GLU353 residues with water molecules, and LEU387 residue. Furthermore, two pi-pi stacking contacts the PHE404 residue with two benzene rings. CDK2 (PDB ID: 1DI8) in complex with DdpMPyPEPhU, many hydrogen bonds participate in this complex, whereas the OH atom interacts with the THR14 residue along two OH atoms; LYS129, ASP145, ASP86, and GLN131 residues with water molecules contact the O atom; two NH atoms interact GLN131, and ASP145 residues; and two N atoms with GLU81, and LEU83 residues. Moreover, a pi-cation contacts the LYS33 residue along the benzene ring, and a pi-pi stacking interact the PHE82 residue with the benzene ring. GR (PDB ID: 1NHZ) in complex with DdpMPyPEPhU, several hydrogen bonds connect with two OH atoms along CYS736, GLN642 residues, and ASP638, GLN738, PRO637 residues with water molecules; the O atom interact the GLN642 residue; the ASN564 residue contacts two NH atoms; and two N atoms interact with the MET604 residue, and ARG611 residue with water molecules. Additionally, three pi-pi stacking contact PHE623 and PHE737 residues along three benzene rings (Fig 9). In all SID analyses, DdpMPyPEPhU demonstrates strong interactions with key residues of the target proteins, indicating its potential to disrupt essential cellular processes involved in cancer progression. These interactions indicate that DdpMPyPEPhU may exert its anticancer effects through multiple mechanisms, including kinase inhibition, hormone signalling disruption, and modulation of metabolic pathways. This multitargeted approach positions DdpMPyPEPhU as a promising candidate for breast cancer treatment. The interaction count is shown with simulation interaction results to provide crucial insights into how our identified compound, DdpMPyPEPhU, can exert its potential efficacy against proteins associated with breast cancer.

### 3.8. Binding free energy analysis

The MM-GBSA was calculated from post-MD simulation trajectories for three different protein structures, identified by their PDB IDs: 1DI8, 1NHZ, and 3ERT. These values are critical for evaluating protein-ligand complexes’ stability and binding affinity, considering the effects of thermal fluctuations during the MD simulations. Hydrogen bond occupancy analysis was conducted to quantify the persistence and stability of key protein–ligand hydrogen bonds over the simulation time. Hydrogen bonds were defined based on standard geometric criteria, including donor–acceptor distance and angle cutoffs, and their occupancies were calculated as the percentage of simulation frames in which each interaction was present. Furthermore, the temporal evolution of binding free energy was evaluated by correlating per-frame MM-GBSA energies with simulation time. This analysis provides insight into the dynamic behavior of ligand binding and confirms the stability of the predicted binding poses under physiological conditions. For the PDB ID- 1DI8 protein in complex with DdpMPyPEPhU, the average binding free energy (dG) is reported as −46.25 kcal/mol, with a standard deviation of 25.00 kcal/mol. This indicates a significant variability in the binding energy across the sampled conformations, suggesting that the system’s interactions fluctuate in strength throughout the simulation. The range of the dG values spans from −79.49 kcal/mol to 3.77 kcal/mol, reflecting both strong binding and occasional weaker interactions at specific time points. The average non-solvent corrected dG (dG(NS)) for 1DI8 is −49.71 kcal/mol, with a standard deviation of 27.04 kcal/mol. The non-solvent corrected dG(NS) range is from −89.17 kcal/mol to 0.20 kcal/mol, confirming the variability observed in the total binding free energy. The non-solvent corrected values suggest that, on average, the protein-ligand interactions are energetically favourable, although there are considerable fluctuations during the MD simulation. For the PDB ID- 1NHZ protein in complex with DdpMPyPEPhU, the average dG is −59.48 kcal/mol, with a standard deviation of 36.36 kcal/mol. This larger standard deviation compared to 1DI8 suggests even more significant fluctuations in binding energy, which may indicate the presence of several conformational states during the simulation, contributing to the wide variation in the binding free energy. The dG range extends from −96.41 kcal/mol to 0.49 kcal/mol, with the negative end indicating strong binding interactions, while the positive value represents weaker states. The average non-solvent corrected dG(NS) is −62.00 kcal/mol, with a 37.89 kcal/mol standard deviation. Similar to the total dG, the non-solvent corrected values for 1NHZ also show a considerable variation, ranging from −100.15 kcal/mol to 0.48 kcal/mol. This again points to a system with highly fluctuating binding interactions, suggesting that the ligand binding is not uniform across the trajectory but may be subject to dynamic changes in conformation and interaction strength. For the PDB ID- 3ERT protein in complex with DdpMPyPEPhU, the average dG is −46.11 kcal/mol, with a standard deviation of 25.60 kcal/mol. This is quite similar to the results for 1DI8, suggesting that the binding interactions are generally favourable but show some degree of fluctuation over time. The dG range is from −78.68 kcal/mol to 2.29 kcal/mol, highlighting both strong binding interactions and occasional weaker binding.

**Fig 10:**
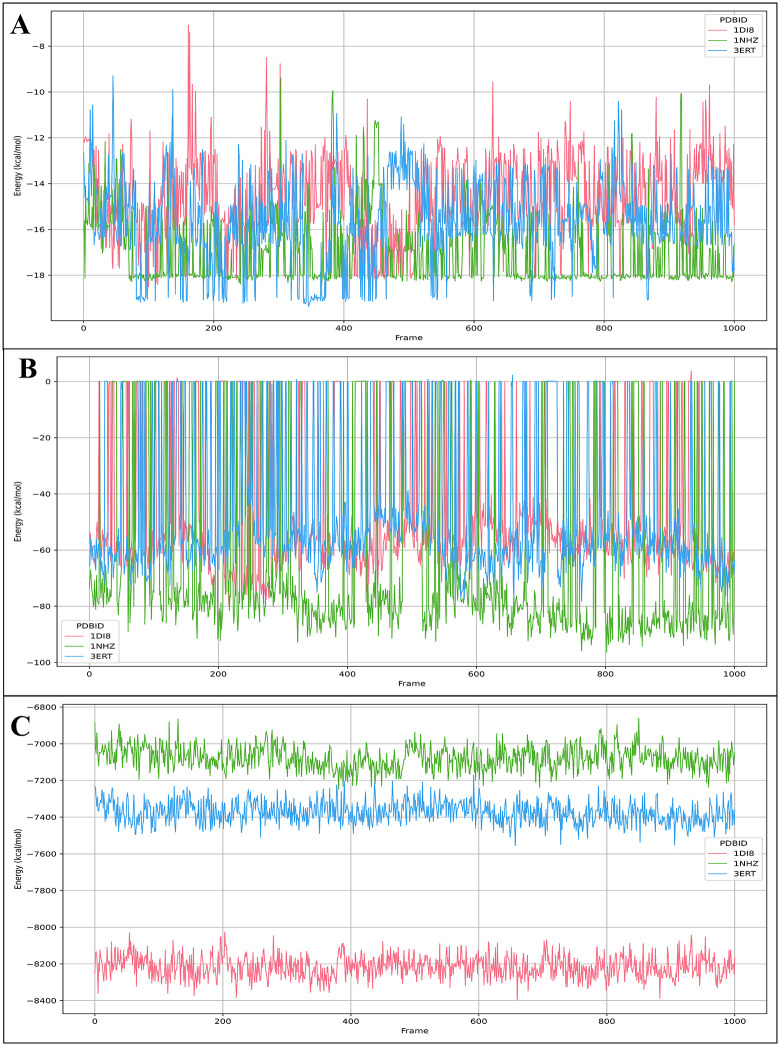
MM-GBSA Results computed on 250 ns (1000 frames) MD Simulations. A) Total Ligand Energy, B) Binding Free Energy and C) Total Complex Energy of DdpMPyPEPhU in complex with CDK2 (PDB ID: 1DI8), Human Estrogen Receptor-α (PDB ID: 3ERT), and GR (PDB ID: 1NHZ), respectively.

States during the simulation. The average non-solvent corrected dG(NS) for 3ERT is −48.77 kcal/mol, with a standard deviation of 27.00 kcal/mol. The non-solvent corrected dG(NS) values range from −83.07 kcal/mol to 1.77 kcal/mol, emphasising the dynamic nature of the protein-ligand interactions. These values show a similar trend to 1DI8, where the ligand binding is predominantly energetically favourable, but the fluctuations in binding strength are still substantial. Furthermore, details for total complex, ligand, and binding free energies are shown for the complete 250 ns simulation in Fig 10. All three proteins (1DI8, 1NHZ, and 3ERT) show significant variability in their binding free energies, both in the total dG values and the non-solvent corrected dG(NS) values. The standard deviations are relatively large, particularly for 1NHZ, suggesting that the systems experience considerable fluctuations in their binding interactions during the 250 ns MD simulations. Despite the variability, the average binding energies for all three proteins are negative, which indicates that the binding interactions are thermodynamically favourable. However, these results also suggest that the systems are dynamic, and the binding strength is not uniform, possibly due to conformational changes or other thermodynamic factors at play during the simulations.

## 4. Discussion

This computer-based work identified a promising new drug candidate, DdpMPyPEPhU, in breast cancer treatment based on the overall multitarget approach. It shows the strength of computational drug repurposing and optimisation technologies in increasing the chemical space of therapeutic discovery and is consistent with recent developments in structure-based drug design [66,67]. A significant strength of breast cancer therapy is the DdpMPyPEPhU multitarget binding profile, since these strategies develop resistance mechanisms and low response to treatment [68,69]. DdpMPyPEPhU emerges as a significantly more promising drug candidate despite not achieving the highest absolute binding affinity values. The in-silico promise of this compound lies not in a single outstanding parameter, but rather in its exceptional balance of multiple critical pharmaceutical properties that collectively determine a molecule’s potential for successful clinical translation. This comprehensive evaluation demonstrates that DdpMPyPEPhU represents a more developable and clinically viable therapeutic option that addresses many of the limitations inherent in the current standard-of-care agents [57,70]. The molecular interaction experiment revealed multiple binding specificities between the three targets, in which DdpMPyPEPhU is hydrogen bonded to important residues, and π-cation binding can be overlapped. This binding specificity implies that the compound might act as a multitargeted inhibitor of several oncogenic signatures, such as cell cycle regulation by inhibiting CDK2 and steroid hormone signalling by glucocorticoid and estrogen receptors, which may have synergistic therapeutic effects [26,71]. Several multitarget strategies have been explored in breast cancer therapy, including CDK inhibitors (e.g., palbociclib), dual ER/HER2 modulators, and kinase–hormone receptor hybrids designed to overcome resistance mechanisms. However, these approaches typically target only two pathways or rely on combination therapy. In contrast, DdpMPyPEPhU was computationally designed to simultaneously modulate ER-α, CDK2, and GR, integrating hormone signaling, cell-cycle control, and stress-response pathways within a single molecular scaffold. This tri-target profile distinguishes DdpMPyPEPhU from existing multitarget inhibitors and supports its potential as a next-generation lead for combating breast cancer heterogeneity and therapy resistance.

The 250 ns MD simulations were a vital confirmation of the results of the static docking by showing the temporal stability of the protein-ligand complexes, which is a limitation of many studies on static docking [72,73]. The RMSD analysis indicated that once equilibration phases were completed, all three complexes were stable with final protein and ligand deviations of 3.15–4.81 and 1.89–3.17, respectively, within a reasonable range of drug-target complexes [74]. RMSF analysis also confirmed that DdpMPyPEPhU continued to interact with key binding site residues during the simulation and that a few important interactions with the key residues’ binding were altered during this time. DdpMPyPEPhU has addressed a key limitation of current breast cancer therapeutics concerning multitarget profiles: most single-target therapies ultimately lead to cellular resistance mechanisms [68]. DdpMPyPEPhU could lead to synergistic therapeutic efficacy while slowing or stopping resistance due to the redundancy of pathways, as it targets cell cycle regulation, hormone signalling, and stress response pathways in breast cancer. MD simulations showed that all three protein-ligand complexes remained stable throughout a 250 ns simulation, with RMSD values within acceptable limits (3.15 to 4.81 Å for proteins; 1.89 to 3.17 Å for ligand), with stable interaction networks of 31–33 residues per individual target complex [72]. ADMET analysis revealed favourable drug-like properties with moderate lipophilicity (QPlogPo/w = 1.555), acceptable oral absorption potential (69.84%), and significant safety margin (no hERG inhibition concerns, QPlogHERG = −4.399). The limited blood-brain barrier permeability (QPlogBB = −2.073) was advantageous and should minimise CNS side effects while accessing peripheral targets. Quite troubling is DdpMPyPEPhU’s low aqueous solubility potential (QPlogS = −3.904), and it may require specialised formulation. Electrostatic potential and molecular electrostatic potentials were detailed using DFT and confirmed thermodynamic stability with favourable free energy (−32.329 kcal/mol) and appropriate electronic properties in biological contexts.

The DFT analysis provided basic information on the electronic structure and thermodynamic stability of DdpMPyPEPhU, fulfilling the need for quantum mechanical corroboration of the chemical mechanistic and modelling hypothesis created using classical molecular mechanics characterisations [75]. In addition to having a secure gas phase energy value (−1221.373659 Hartrees) and positive vibrational frequencies, which indicated stability, an important confirmation that the compound would be stable and not readily undergo decomposition. The HOMO-LUMO energy gap analysis indicates that DdpMPyPEPhU has appropriate electronic properties likely to make interactions with biological macromolecules. The significant dipole moment of 9.2975 Debye indicates considerable polarity, allowing the compound to form hydrogen bonding and electrostatic-type interactions with protein targets easily [76]. The calculated thermodynamic parameters at physiological conditions indicate a favourable change in free energy (−32.329 kcal/mol), indicating the compound is stable under biological conditions.

WaterMap analysis of the ligand binding site in four structures revealed multiple sites of water displacement ranging from ΔG < 0 kcal/mol to +8.54 kcal/mol for “high-energy” displacing water. These represent clear optimisation opportunities to enhance binding affinity through hydrophobic substitutions [77]. Clearly, the computational predictions and assessments warrant experimental validation through further investigation. DdpMPyPEPhU’s balanced interaction profile that encapsulates moderate binding strength and multitargeting provides it the potential to be a better therapeutic candidate than single targeting approaches currently used for breast cancer treatment [59,78]. It targets cell cycle regulation by CDK2, steroid hormone signalling by ER-α, and stress responses by GR at the same time, thereby possibly exerting a synergistic anticancer effect while diminishing the chance of developing drug resistance due to the redundancy of pathways to target. Overall, DdpMPyPEPhU will directly target multiple pathways to obtain maximum anticancer activity. This polypharmacological approach could complement the growing interest in precision medicine and its recognition that cancer is a multi-pathway disease requiring treatment from multiple pathways [79,80]. The WaterMap analysis provides actionable insights into the rational optimization of DdpMPyPEPhU. High-energy hydration sites identified near the ligand binding regions of ER-α, GR, and CDK2 were displaced upon ligand binding, indicating entropically and enthalpically favorable interactions. These sites define chemically addressable hot-spots where incorporation of polar substituents or hydrogen-bonding moieties could further improve affinity and selectivity. Importantly, several of these hydration hot-spots were conserved across all three targets, suggesting that structure-guided modification at these positions may enhance multitarget potency rather than compromise it. Thus, WaterMap not only validates the binding mode of DdpMPyPEPhU but also provides a rational framework for future lead optimization.

This computational investigation has several limitations that should be stated and recognised. First, the analysis relied wholly on *in silico* predictions that were not experimentally validated or substantiated. Reasonably, computational binding affinities may not represent the biological milieu with complexities such as protein flexibility, allostery, and the cellular context [81,82]. The present binding poses did not consider ligand-water interactions, which present possible opportunities for optimisation that were not considered in this study. Furthermore, the drug enumeration process only considered specific chemical scaffolds from FDA-approved drugs, presumably leaving out other structural classes that could lead to better candidates. The ADMET predictions were comprehensive yet based on computational models that will not represent all features of human pharmacokinetics, particularly drug-drug interactions, variations in metabolism, and differences between populations [83]. The MM-GBSA analysis of protein-ligand complexes for PDB IDs 1DI8, 1NHZ, and 3ERT shows that these systems are relatively stable but exhibit significant fluctuations in binding free energy during 250 ns MD simulations. The average binding free energies (dG) for all three proteins are negative, indicating overall thermodynamic favourability of ligand binding. However, substantial standard deviations, especially for 1NHZ, highlight the dynamic nature of these interactions. For 1DI8, the binding free energy fluctuated between −79.49 kcal/mol and 3.77 kcal/mol, suggesting both strong and weak binding states across the simulation. Similarly, 1NHZ showed a wider range, with dG values from −96.41 kcal/mol to 0.49 kcal/mol, further supporting the idea of fluctuating binding strength. 3ERT displayed a comparable range, with values between −78.68 kcal/mol and 2.29 kcal/mol, suggesting occasional weaker binding states. These variations are also reflected in the non-solvent corrected binding energies (dG(NS)), where the range spans from −100.15 kcal/mol to 0.48 kcal/mol for 1NHZ, indicating that solvent effects may play a significant role in modulating binding. Despite the large fluctuations, all three complexes’ negative average dG values point to stable binding overall, with dynamic transitions between different conformational states. The data suggest that these protein-ligand complexes are not static but undergo continuous fluctuations, highlighting their dynamic nature and potential for adaptable binding interactions.

DdpMPyPEPhU represents a chemically novel scaffold integrating pyrimidine, phenylurea, and heteroaromatic fragments. Each component is synthetically accessible using commercially available precursors through standard urea-coupling and substitution reactions, indicating that the compound is not only computationally promising but also chemically feasible. The favourable computational data for DdpMPyPEPhU require rapid progression into experimental testing through in vitro binding studies, cytotoxicity studies, and selectivity profiles in breast cancer, with the potential to develop the use of normal breast cells as the comparative cell line. Structure-activity relationship studies should pursue the additional development of structure physicochemical properties based on the potential energy displacement of high-energy water sites while maintaining the desirable multitarget binding profile. Additionally, the formulations should address solubility and could employ a novel pharmaceutics degree of technology improvements related to formulation via solid dispersions, nanoparticles or co-crystal approaches [84]. Given the finding of a DdpMPyPEPhU multitarget to illustrate a normal tissue safety profile, the following steps should explore the potential for NS3 synergistic binding along with other breast cancer therapies and further determine the activity of DdpMPyPEPhU in drug-resistant cancer cell lines related to CDK inhibitors or hormone therapies. This compound’s balanced interaction profile and moderate binding energies could yield a better therapeutic index than some current clinical targets, which may have higher potency but with more toxicity [85]. DdpMPyPEPhU should be defined as an up-and-coming next-generation drug candidate that achieves in-silico promising overall pharmaceutical characteristics compared to currently used FDA drugs for breast cancer. Rather than competing on the single dimension of binding affinity, where it shows respectable but not exceptional performance, DdpMPyPEPhU excels in the multidimensional space that determines drug success, combining adequate potency with exceptional solubility, excellent drug-likeness, and stable receptor engagement. Future medicinal chemistry endeavours should maintain the multitarget drug action while optimising physicochemical properties (predominantly aqueous solubility and metabolic stability) to develop this approach towards preclinical testing and ultimately clinical applications.

## 5. Conclusion

Although the U.S. FDA continues to approve new drugs, their high cost, limited accessibility, and uncertain effectiveness remain major concerns for the public. Additionally, potential side effects raise significant safety questions. Many studies fail to provide clear conclusions on whether these drugs effectively target multiple proteins or cancer types. This study explores the implications for drug enumeration toward single capsule-based drug design and development against breast cancer therapy. We found that complete readiness improves drug configuration through docking. Further, the studies extended automated enumeration to generate novel candidates and docked them again in the same grid, which led to identifying DdpMPyPEPhU as a novel drug candidate against breast cancer. The consistency of binding behavior observed across docking and long-timescale 250 ns MD simulations, combined with favorable hydration thermodynamics and electronic properties, underscores the robustness of the proposed compound. Importantly, this multitarget strategy offers a potential advantage in mitigating drug resistance commonly associated with single-target therapies. After getting the best docking poses, we performed the MM-GBSA analysis and then extended it for 250 ns MD simulation studies that confirmed the stability of the complexes, and the ligand stability was analysed along with the DFT analysis. The complex analysis of protein preparation, ligand, multilevel docking, MM-GBSA, MD simulation, and DFT analysis leads us to understand the theoretical behaviours of DdpMPyPEPhU against breast cancer. Our novel compound simultaneously targets key oncogenic pathways – including cell cycle regulation via CDK2, hormone signalling through estrogen and glucocorticoid receptors, and stress response pathways, that produce synergistic anticancer effects while reducing the likelihood of resistance development. Its docking and 250 ns MD simulations demonstrate stable and specific binding across multiple targets, supported by acceptable RMSD values and sustained residue interactions over time. ADMET analyses further highlight its moderate lipophilicity, good oral absorption potential, minimal CNS penetration, and favourable safety margins. Although improvements in aqueous solubility may be required, the compound’s overall physicochemical and pharmacological profile suggests it could offer better therapeutic outcomes and a computationally predicted safety profile compared to current standard treatments. These findings provide strong computational evidence supporting the therapeutic relevance of the proposed scaffold and lay the groundwork for subsequent experimental validation and lead optimization studies. However, its extensive characterisation, followed by experimental validation, is required before further use.

## Supporting information

S1 File DdpMPyPEPhU_Supplementary_File. (XLSX)

## Abbreviations

DdpMPyPEPhU: 1-((R)-2,3-dihydroxypropyl) −3-(3-((R)-1- (5-methyl-1H-pyrrolo [2,3-b] pyridin-3-yl) ethyl) phenyl) urea
MDS: Molecular Dynamics Simulation
DFT: Density Functional Theory
MM-GBSA: Molecular Mechanics\Generalised Born Surface Area
WHO: World Health Organisation
3D: Three-Dimensions
Å: Angstrom
VSW: Virtual Screening Workflow
HTVS: High-Throughput Virtual Screening
SP: Standard precision
XP: Extra Precise
CSV: Comma separated Values
ADMET: absorption, distribution, metabolism, excretion, and toxicity
HOMO: highest occupied molecular orbital
LUMO: lowest unoccupied molecular orbital
SID: simulative interaction diagram
SCF: self-consistent field
RMS: root mean square
RMSD: root mean square deviation
RMSF: root mean square fluctuation
FDA: Food & Drug Administration
SPC: simple point charge
